# MiR-10a and HOXB4 are overexpressed in atypical myeloproliferative neoplasms

**DOI:** 10.1186/s12885-018-4993-2

**Published:** 2018-11-12

**Authors:** Pierre-Yves Dumas, Olivier Mansier, Valerie Prouzet-Mauleon, Junji Koya, Arnaud Villacreces, Philippe Brunet de la Grange, Damien Luque Paz, Audrey Bidet, Jean-Max Pasquet, Vincent Praloran, Franck Salin, Mineo Kurokawa, François-Xavier Mahon, Bruno Cardinaud, Eric Lippert

**Affiliations:** 1 CHU de Bordeaux, Hématologie Clinique et Thérapie Cellulaire, F-33000 Bordeaux, France; 2 INSERM U1035, Université de Bordeaux, Bordeaux, France; 3 INSERM U1218, Université de Bordeaux, Bordeaux, France; 4 CHU de Bordeaux, Laboratoire d’Hématologie, F-33000 Bordeaux, France; 5 Department of Hematology and Oncology, Graduate School of Medicine, University of Tokyo, 7-3-1, Hongo, bunkyo-ku, Tokyo, 113-8655 Japan; 6 Etablissement Français du Sang - Aquitaine Limousin, Laboratoire R&D d’Ingénierie Cellulaire, Université de Bordeaux, Bordeaux, France; 7 INRA, Plateforme Génome Transcriptome de Bordeaux, BIOGECO, UMR 1202, F-33610 Cestas, France; 8 Institut Bergonié, Bordeaux, France; 9 Bordeaux Institut National Polytechnique, F-33000 Bordeaux, France; 10 CHRU de Brest, Service d’Hématologie Biologique et INSERM U1078, Université de Bretagne Occidentale, Brest, France; 11 CHRU d’Angers, Laboratoire d’Hématologie, Angers, France

**Keywords:** Atypical myeloproliferative neoplasms, HOXB4, miR-10a, DNMT3A, Epigenetic

## Abstract

**Background:**

Atypical Myeloproliferative Neoplasms (aMPN) share characteristics of MPN and Myelodysplastic Syndromes. Although abnormalities in cytokine signaling are common in MPN, the pathophysiology of atypical MPN still remains elusive. Since deregulation of microRNAs is involved in the biology of various cancers, we studied the miRNome of aMPN patients.

**Methods:**

MiRNome and mutations in epigenetic regulator genes *ASXL1*, *TET2*, *DNMT3A*, *EZH2* and *IDH1*/*2* were explored in aMPN patients. Epigenetic regulation of miR-10a and *HOXB4* expression was investigated by treating hematopoietic cell lines with 5-aza-2’deoxycytidine, valproic acid and retinoic acid. Functional effects of miR-10a overexpression on cell proliferation, differentiation and self-renewal were studied by transducing CD34^+^ cells with lentiviral vectors encoding the pri-miR-10a precursor.

**Results:**

MiR-10a was identified as the most significantly up-regulated microRNA in aMPN. MiR-10a expression correlated with that of *HOXB4*, sitting in the same genomic locus. The transcription of these two genes was increased by DNA demethylation and histone acetylation, both necessary for optimal expression induction by retinoic acid. Moreover, miR-10a and *HOXB4* overexpression seemed associated with *DNMT3A* mutation in hematological malignancies. However, overexpression of miR-10a had no effect on proliferation, differentiation or self-renewal of normal hematopoietic progenitors.

**Conclusions:**

MiR-10a and *HOXB4* are overexpressed in aMPN. This overexpression seems to be the result of abnormalities in epigenetic regulation mechanisms. Our data suggest that miR-10a could represent a simple marker of transcription at this genomic locus including *HOXB4*, widely recognized as involved in stem cell expansion*.*

**Electronic supplementary material:**

The online version of this article (10.1186/s12885-018-4993-2) contains supplementary material, which is available to authorized users.

## Background

Mixed Myeloproliferative Neoplasm/myelodysplastic syndrome (MPN/MDS) comprise atypical Chronic Myeloid Leukemia (aCML), Chronic MyeloMonocytic Leukemia (CMML), Juvenile MyeloMonocytic Leukemia and unclassified MPN/MDS (uMPN/MDS). Atypical CML and uMPN/MDS, hereafter referred to as atypical MPN (aMPN) often have clinical presentation reminiscing of Chronic Myelogenous Leukemia (CML) with hyperleukocytosis, mainly composed of mature and immature granulocytes, co-existing with myelodysplastic features. In CML, and in most classical MPN, hyperproliferation can be related to abnormalities of tyrosine kinases (ABL1 in CML, JAK2 in many other MPN) or other proteins involved in the cytokine signaling pathways (MPL, LNK, CBL*,* etc.*).* Recently, mutations in *CSF3R* and/or *SETBP1* were reported as involved in the hyperproliferation in aMPN. However, not all aMPN patients carry mutations in these genes, and the leukemogenic mechanisms are not fully understood, especially regarding the dysplastic features [1, 2].

MicroRNAs (miRNAs) are small non-coding RNAs that bind to specific mRNA targets leading to translational repression and/or mRNA cleavage. MiRNAs play important roles in various cell processes, including differentiation, proliferation, and apoptosis. Mature miRNAs are processed from hairpin-shaped precursors that are encoded by dedicated genes or by intronic sequences of other genes [3]. *HOX* clusters encode highly conserved transcription factors characterized by the presence of a homeobox domain capable of binding to DNA, determinant for correct anterior to posterior patterning of the body axis during development [4, 5]. Several types of non-coding RNAs have been retained within the *HOX* clusters over the course of evolution, including miR-10 and miR-196 families [6, 7]. MiR-10a is located between *HOXB4* and *HOXB5* genes in mammals. MiR-10 family members have also been found to target *HOX* transcripts in several species, probably playing an important role during development.

Several human *HOX* transcripts have been experimentally validated as miR-10a targets, including *HOXA1, HOXA3, HOXD10, HOXB1* and *HOXB3* [8–10]. Aside *HOX* transcripts, miR-10a has also been shown to regulate *USF2, HDAC4, SFRS1* and *NCOR2* [11–14]. In the hematopoietic system, miR-10a is expressed in CD34^+^ stem/early progenitor cells, and in vitro differentiation of CD34^+^ cells into megakaryocytes is marked by a decreased level of both miR-10a and miR-10b [8]. Accordingly, levels of miR-10a are markedly higher in hematopoietic stem cells than in peripheral blood lymphocytes [15]. Several articles have reported a deregulation of miR-10 family members in human cancers, including several types of myeloid malignancies [11, 16, 17]. For example, miR-10a has been found downregulated in CML [11] but upregulated in *NPM1*-mutated acute myeloid leukemias (AML) [16, 18–20]. Both miR-10a and miR-10b have also been found upregulated in some neurological tumors [21], in hepatocellular carcinomas [22] and in pancreatic cancers [23]. However, the precise mechanism of miR-10a oncogenic potential remains unclear [24].

The miR-10 family is encoded in the mammalian *HOXB* cluster and little is known about the mechanisms that govern the regulation of miR-10a expression. MiR-10a being co-regulated with *HOX* genes, an epigenetic control could regulate their expression since methylation of CpG islands in promoter regions plays a critical role in the expression of various genes including miRNAs [9]. In this study, we found miR-10a as the most differentially dysregulated miRNA in aMPN when compared to CML, leukocytes of reactive states or healthy donors. We then showed that miR-10a expression requires opening of the chromatin (DNA demethylation, histone deacetylase inhibition) for optimal up-regulation, especially in response to retinoic acid (RA) stimulation. This is coherent with our demonstration of a higher miR-10a/*HOXB4* expression in patients with *DNMT3A* mutation. Finally, we did not observe any functional effect of miR-10a overexpression in normal hematopoietic progenitor proliferation, differentiation or self-renewal. These results support the hypothesis whereby miR-10a could represent a marker of *HOXB4* transcription, without a specific leukemogenic function, at least in myeloid cells.

## Methods

### Patient biological samples

Samples were obtained from patients of the University Hospitals of Bordeaux, Brest and Angers, who gave written informed consent for the use of remaining nucleic acids for research. They were diagnosed with aCML, uMPN/MDS, *NPM1*-wild-type AML, primary myelofibrosis (PMF), CMML or CML. Reactive hyperleukocytosis (RHL) corresponded to inflammatory states due to major burns or surgical operation. Written informed consent was obtained from all patients in accordance with the Declaration of Helsinki, allowing the collection of clinical and biological data in an anonymized database, registered at the Commission Nationale de l’Informatique et des Libertés under N°1777604.

### DNA and RNA extraction

DNA and RNA were obtained from total peripheral blood leucocytes (PBL) for aMPN, CML, RHL and healthy donors, or bone marrow mononuclear cells (BMMC) for AML.

### Microarray hybridization

Cyanine-3 (Cy3) labeled miRNA was prepared from 0.2 μg RNA using the miRNA complete labeling kit version 2.2 (Agilent Technologies). For each sample, the labeled miRNAs were hybridized overnight at 55 °C onto Human miRNA Microarray V2 (Agilent Technologies). Normalized data for all samples have been deposited in NCBI Gene Expression Omnibus and are accessible through GEO Series accession number GSE75666.

### Genotyping

10 ng DNA were used for highly multiplex amplification of *DNMT3A* (exons 15–23), *IDH1/2* (exon 4), *ASXL1* (exon 12), *TET2* and *EZH2* (whole coding region) with an AmpliSeq™ panel (Thermo Fischer Scientific) before sequencing on an Ion Torrent PGM (Life Technologies) with 314 chips.

### Culture and pharmacological agents

For pharmacological experiments, AML cell lines were used as model of myeloid diseases. U937 (monocytic cell line), KG1a (promyeloblast cell line) and OCI-AML3 (myelomonocytic cell line) (DSMZ) were treated by 5-aza-2’deoxycytidine (DEOX) (Sigma-Aldrich) and/or valproic acid (VPA, Calbiochem) and/or retinoic acid (RA). K562 cells (erythroleukemia cell line) were used for proliferation assays. CD34^+^ cells were cultured in Stem Span SFEM (Stemcell Technologies), MethoCult H4534 Classic without EPO (Stemcell technologies) or on MS5 cells in Myelocult H5100 (Stemcell Technologies). When indicated, transduced cells were selected by puromycin treatment (Sigma-Aldrich).

### Lentiviral vectors

For miR-10a over-expression, the pri-miRNA precursor was cloned into plasmids carrying a puromycin resistance or a GFP gene, under the control of a MND promoter. Wild Type and R882H mutated *DNMT3A* cDNA were obtained from pMYs vectors previously reported [25] and re-cloned into lentiviral vectors carrying a puromycin resistance gene, under the control of a EF1alpha promoter. Lentiviral vectors were produced by the vectorology platform of the University of Bordeaux.

### Real-time qPCR

For *HOXB4* mRNA quantification, complementary DNA (cDNAs) were synthetized from 1 μg of RNA, using the First Strand cDNA synthesis kit (Roche®, Meylan, France) according to manufacturer’s instructions. The real time quantitative PCR (qRT-PCR) was performed using Brilliant SYBR® Green QPCR kit on a Mx3005P thermocycler (Stratagene®, Massy, France). For miR-10a quantification, 500 ng of total RNA was reverse-transcribed using the 1st-Strand miRNA cDNA Synthesis kit (Stratagene®) according to manufacturer’s instructions. Quantitative RT-PCR was performed using the High-Specificity miRNA qPCR Core Reagent Kit (Stratagene®).

### Flow cytometry analysis

Cellular suspensions were incubated with different panels of fluorochrome-labelled antibodies and analyzed on a FACS CANTO II (Beckton Dickinson). Data were analyzed with FacsDiva software.

### Mouse transplantation

Three sets of experiments were conducted, each with 10 female NSG (NOD.Cg-Prkdcscid Il2rgtm1Wjl/SzJ) mice for each condition. Mice were bred under specific pathogen-free conditions and experiments were performed in the animal housing facility of the University of Bordeaux in conformity with the rules of the Institutional Animal Care and Use committee (Ministerial approval number 00048.2). CD34^+^ cell engraftment protocol is detailed in the Additional file 1: Supplementary Material.

### Statistical analysis

Student’s t-test and Mann and Whitney test have been performed using GraphPad Prism. More details about these methods are available in the Additional file 1: Supplementary Material.

## Results

### MiR-10a is upregulated in aMPN compared to CML, reactive hyperleukocytosis and healthy donors

**Fig. 1:**
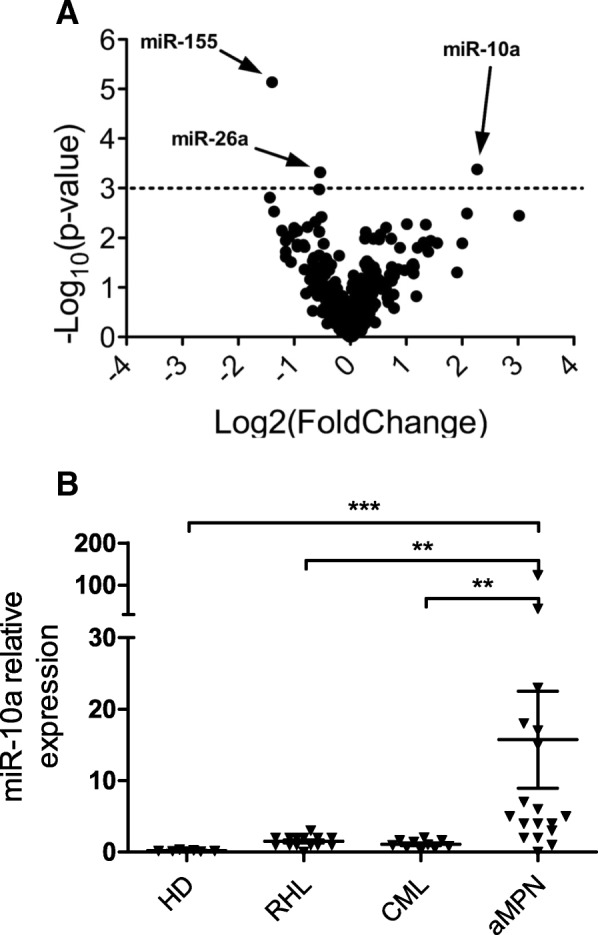
MiR-10a is differentially expressed in the aMPN, CML**,** RHL and HD groups. **a** Blood leukocyte RNA from patients with aMPN (*n* = 18) or CML (*n* = 10) were analyzed on an Human miRNA Microarray V2 (Agilent Technologies). The volcano plot represents the analysis of differentially expressed miRNAs in aMPN as compared to CML: the expression difference in miRNA expression is plotted on the x axis, and *p*-value significance is plotted on the y axis (−log10 scale). MicroRNAs that are found differentially expressed (*p* < 0.001) are arrow-pointed. **b** qRT-PCR quantification of miR-10a in peripheral blood cells from healthy donors (HD, *n* = 7), donors with reactive hyperleukocytosis (RHL, *n* = 14), patients with CML at diagnosis (*n* = 10) and patients with aMPN at diagnosis (n = 18) patients. The graph shows relative expression of gene by disease with mean ± SEM

In order to discover micro-RNAs specifically dysregulated in aMPN compared to CML with similar blood leukocyte and differential counts, a total of 18 aMPN (Patients 1 to 18 in Additional file 2: Table S1) and 10 CML samples were hybridized on Agilent miRNA microarrays. An univariate analysis identified genes that were differentially expressed in the two groups. Using a stringent significance level for each univariate test of 0.001, 3 miRNAs were characterized as differentially expressed in aMPN versus CML (miR-155: *p* = 7.3 × 10^− 6^, FDR = 0.006; miR-10a: *p* = 4.2 × 10^− 4^, FDR = 0.132 miR-26a: *p* = 4.8 × 10^− 4^, FDR = 0.132, Fig 1a). These 3 miRNAs were also found as differentially expressed by a SAM (significance analysis of microarrays) using a target proportion of false discovery of 0.05 (not shown). Among them, miR-10a had the highest fold-change (4.8). We then focused our analyses on the overexpression of this microRNA. In CML patients, miR-10a expression has been reported lower than in healthy donors [11]. We confirmed microarrays results by comparing leukocytes from aMPN (*n* = 18) to 10 patients with CML at diagnosis, 14 reactive hyperleukocytosis (RHL) and 7 healthy donors (HD) by specific RT-qPCR. The significant overexpression of miR-10a in the aMPN group compared to the three others was confirmed (Fig. 1b).

### MiR-10a and *HOXB4* overexpression depends on epigenetic regulation

**Fig. 2:**
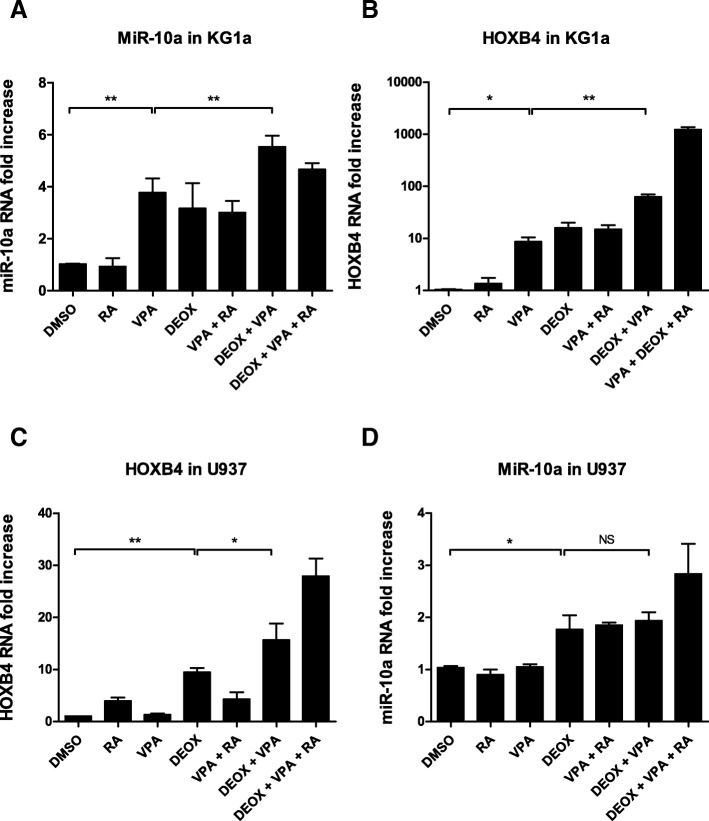
Expression of HOXB4 and miR-10a in cell lines according to treatment by 5-aza-2’deoxycytidine (DEOX), valproic acid (VPA), and retinoic acid (RA). KG1 (**a**, **b**) and U937 (C, D) were treated with 2 μM 5-aza-2’deoxycytidine (DEOX) and/or 1 mM valproic acid (VPA) for 48 h and/or 2 μM retinoic acid (RA) for 4 h. After RNA extraction, quantitative RT-PCR measured the relative expression miR-10a with *RNU6–1* as a control gene (**a**, **d**) and of *HOXB4* with *TUBA1C* and *RPLP0* both used as control genes (**b**, **c**). The graphs show fold induction of gene expression by treatment over DMSO control with mean ± SEM (*n* = 3). *, *p* < 0.05; **, *p* < 0.01; ***, p < 0.001; NS, non-significant

Since mutations in epigenetic regulators have been described in aMPN, and because demethylating agents have proved efficient in treating myelodysplastic syndromes, we used myeloid cell lines to investigate whether miR-10a expression could be regulated by epigenetic mechanisms in hematopoietic cells. MiR-10a sitting immediately upstream of *HOXB4*, we studied the expression of both RNAs. In KG1a cells, treatment by valproic acid (VPA) at dose of 1 mM for 48 h induced an overexpression of miR-10a with a stronger effect when VPA was combined with 2 μM 5-aza-2’deoxycitidine (DEOX). On the contrary, 2 μM retinoic acid (RA) alone or in combination was inefficient at increasing miR-10a expression (Fig. 2a). Regarding *HOXB4*, DEOX alone, and to a larger extent in combination with VPA, also increased its expression. In contrast to miR-10a, the addition of RA had an additional effect, suggesting that the optimal increase in *HOXB4* expression due to RA requires an optimal chromatin opening (Fig. 2b). This epigenetic regulation was variable depending on the AML cell line: in *NPM1*- and *DNMT3A*-wild type U937 cells, treatment by 1 mM VPA alone did not induce any modification in *HOXB4* or miR-10a levels whereas 2 μM DEOX alone did induce overexpression of both transcripts. This effect was potentiated by VPA for *HOXB4*, but not for miR-10a, and combination of the three drugs induced a 1.5 to 3-fold overexpression compared to DEOX alone, for both transcripts (Fig. 2c-d). Finally, in the *NPM1*- and *DNMT3A*-mutated cell line OCI-AML3, treatment with 2 μM DEOX did not induce *HOXB4* overexpression, whereas 1 mM VPA induced a 3 to 4-fold overexpression. This effect was not potentiated by 2 μM RA or 2 μM DEOX, but the association of the three drugs induced an 8-fold overexpression of *HOXB4*, significantly superior to that observed with VPA alone (Additional file 3: Figure S1A). These data suggest that the level of basal expression of miR-10a and *HOXB4* depends on epigenetic regulation in these myeloid cell lines (Additional file 3: Figure S1B) and their drug sensitivity profile seems to be variable depending on their mutational status.

### MiR-10a and *HOXB4* co-expression depends on *DNMT3A* mutational status

**Fig. 3:**
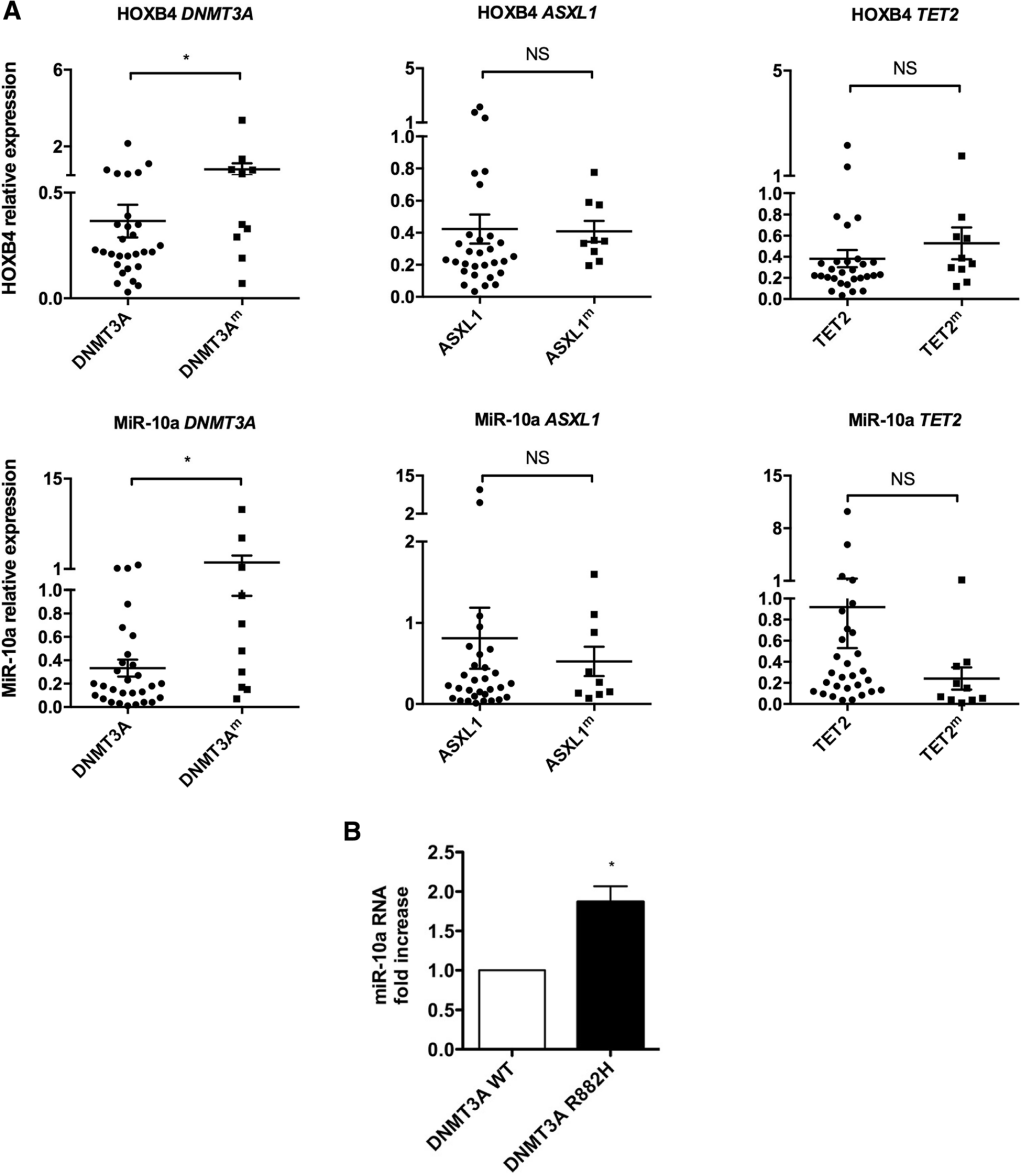
Expression of *HOXB4*/miR-10a according to epigenetic modifiers mutational status. **a** NGS sequencing of *TET2*, *ASXL1*, *EZH2*, *DNMT3A* and *IDH1/2* was performed on genomic DNA from the cohort of hematological malignancies described in Additional file 2: Table S2. Relative expression of *HOXB4* and miR-10a were measured by qRT-PCR. **b** CD34^+^ primary hematopoietic cells purified from cord blood were transduced with a lentivirus allowing the expression of WT or R882H mutated DNMT3A and grown in complete medium containing SCF, TPO and Flt3-L. After 3 days of selection in the presence of puromycin (1 μg/mL), the expression of miR-10a was assessed by qRT-PCR. Results are expressed as the expression of miR-10a normalized on *RNU6–1* expression and compared to the WT condition (n = 3). *, p < 0.05; **, p < 0.01; NS, non-significant

Having established the epigenetic regulation of *HOXB4*/MiR-10a expression, we wondered whether the expression level of these genes may correlate with the mutational status of epigenetic modifiers frequently mutated in hematological malignancies. Six epigenetic regulators were sequenced in 39 patients with myeloid malignancies (Additional file 2: Table S2) and levels of HOXB4 and miR-10a were measured. We observed a significantly higher *HOXB4* and miR-10a expression in patients with *DNMT3A* mutations while *TET2*, *ASXL1*, *IDH1/2*, and *EZH2* mutational status did not influence *HOXB4* nor miR-10a expression level (Fig. 3a and data not shown). Although this validation cohort did not include aMPN patients, taken together our data strengthen the evidence of an epigenetic regulation of miR-10a and *HOXB4*. In order to confirm the impact of *DNMT3A* mutational status on miR-10a expression, we transduced primary hematopoietic CD34^+^ cells with lentiviruses carrying the wild type or the R882H mutant *DNMT3A* cDNA. As previously reported for *HOXB4* [25], the expression of miR-10a was significantly higher in the R882H DNMT3A compared to the wild type DNMT3A condition (Fig. 3b).

### Absence of functional effect of miR-10a overexpression on proliferation, differentiation or self-renewal of CD34^+^ hematopoietic progenitors

**Fig. 4:**
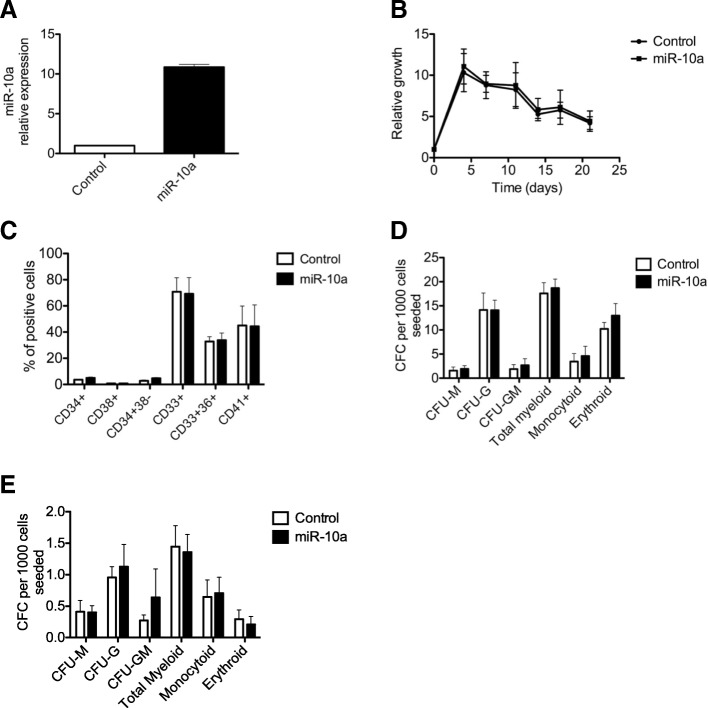
Functional consequences of miR-10a overexpression in hematopoietic stem progenitor cells. CD34^+^ primary hematopoietic cells purified from cord blood were transduced with a lentivirus expressing the pri-miR-10a precursor or the empty vector and grown in complete medium containing SCF, TPO and Flt3-L. **a** Efficient overexpression of miR-10a was assessed by qRT-PCR on CD34^+^ cells 4 days after transduction. Results are expressed as the expression of miR-10a in one representative experiment, normalized on *RNU6–1* expression and compared to empty vector condition (*n* = 2). **b** Cells were counted at different times after transduction. Results are expressed as fold increase after stimulation. The graph represents the mean values of 2 independent experiments. **c** After 7 days of culture, differentiation markers expression was assessed by flow cytometry. The graph represents the percentage of positive cells in one representative experiment performed in duplicate (n = 2) (**d**). After 7 days of liquid culture, cells were implanted in semi-solid medium in presence of EPO (for erythroid colonies) or G-CSF (for myeloid colonies). The graph represents the mean colony number for 1000 cells seeded obtained in 2 independent experiments. **e** Stem cell renewal was assessed by culturing transduced normal CD34^+^ cells on MS5 stromal cells for 5 weeks. Clonogenic capacity was then assessed in semi-solid medium in presence of EPO (for erythroid colonies) or G-CSF (for myeloid colonies). Colonies were counted after 2 weeks. Graph shows mean colony number for 1000 cells seeded of 4 independent experiments

**Fig. 5:**
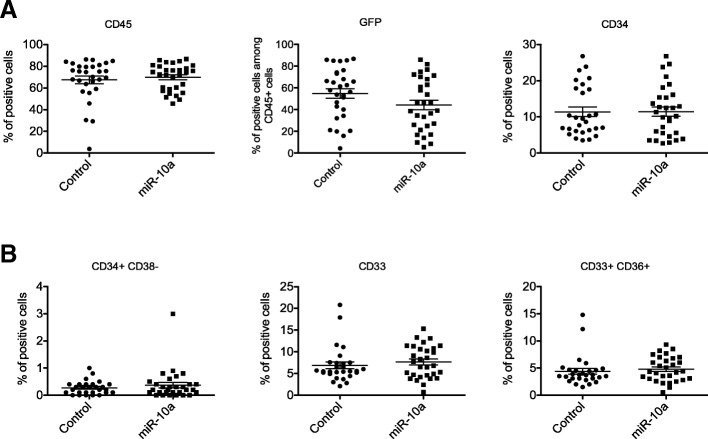
Effect of miR-10a overexpression on hematopoietic stem cell xenotransplantation into NSG mice. CD34^+^ cells were transduced with lentiviral vectors encoding pri-miR-10a and a GFP marker or a control (empty vector), then transplanted into myeloablated mice. **a** After 12 weeks, the bone marrows were harvested and differentiation marker expression on human transduced cells was assessed by flow cytometry. **b** Each dot represents the percentage of positive cells observed in one mouse, among GFP^+^ cells for CD34, CD38, CD33 and CD36. Results of 3 independent experiments are grouped

The role of *HOXB4* in stem cell expansion in hematopoiesis being well documented [26–28], we explored the role of miR-10a overexpression in the main functions of hematopoietic cells relevant to leukemogenesis. Firstly, we transduced K562 and U937 cell lines with a vector carrying the pri-miR-10a precursor and evaluated the impact of miR-10a overexpression on cell proliferation. Despite an overexpression by a mean factor of 5 and 45 for K562 and U937 respectively, we did not observe any effect on cell proliferation (Additional file 3: Figure S2). To evaluate cellular properties more related to leukemogenesis such as differentiation or self-renewal, we used the same construction to force overexpression of miR-10a in human primary CD34^+^ cells. Impact on late hematopoiesis was analyzed by culturing the cells for 3 weeks in liquid medium. Despite a mean 13.8-fold increased expression as assessed by qRT-PCR (Fig. 4a), similar to that observed in aMPN patients, miR-10a overexpression did not alter cell proliferation (Fig. 4b). At days 7, 14 and 21, differentiation was evaluated by flow cytometry and clonogenic capacities. We did not observe any impact of miR-10a overexpression on the level of granulocytic, erythroblastic or megakaryocytic differentiation markers (Fig. 4c), nor on the clonogenic capacities of primary cells (Fig. 4d). Similar results were observed when CD34^+^ cells were transfected with a synthetic miR-10a precluding any abnormal processing of the pri-miR-10a precursor (Additional file 3: Figure S3). Effects of miR-10a overexpression on more immature compartments of hematopoiesis were assessed in vitro by cultures on a stromal cell layer (LTC-IC assay). After 5 weeks, clonogenic capacities of transduced cells were evaluated. As in liquid culture experiments, miR-10a overexpression did not alter differentiation nor self-renewal of primary cells (Fig. 4e). Finally, the roles of miR-10a in hematopoiesis were analyzed in xenograft experiments. CD34^+^ cells were transduced with the pri-miR-10a or the empty vector both co-expressing GFP and injected into the retro-orbital sinus of NSG mice. Twelve weeks after engraftment, bone marrow cells were analyzed by flow cytometry. MiR-10a was efficiently over-expressed with a mean 5.6-fold increased expression compared to controls. Meanwhile, we did not observe any impact on engraftment efficiency, self-renewal (determined by the proportion of human CD34^+^ cells among GFP^+^ cells), cell proliferation (as assessed by the proportion of GFP^+^ cells among human CD45^+^ cells) (Fig. 5a) nor cell differentiation (Fig. 5b). Effects on self-renewal were further analyzed by secondary engraftment experiments. Once again, we did not observe any effect of miR-10a over-expression on the level of the different markers compared to controls (Additional file 3: Figure S4).

## Discussion

Atypical MPN are characterized by the association of proliferative and myelodysplastic features. The discovery of mutations in genes involved in cytokine signaling, mainly *CSF3R* and *SETBP1*, explains the proliferative part in some patients. However, little is known of the possible involvement of microRNA deregulation in these diseases. Yet, microRNA overexpression has been pointed as a potential driver of oncogenesis in several cancer models, leading to the concept of “oncomir” [29]. For these reasons, we studied the miRNome of peripheral leukocytes in patients with aMPN and compared it to patients with CML, a typical myeloproliferative neoplasm, RHL, a non-malignant myeloid expansion condition, and healthy donors. MiR-10a was the most differentially expressed microRNA in aMPN compared to CML. Moreover, it was found upregulated compared to all control groups. Even though miR-10a has been found expressed at higher levels in immature cells [8, 15], the difference we observed was not due to an over-representation of immature cells in the aMPN group since leukocyte differentials were similar in RHL and CML groups and there was no correlation between miR-10a levels and blast or immature granulocyte counts.

Interestingly, miR-10 family members are included within *HOX* clusters and a co-regulation between miR-10a and *HOXB4* has been reported during development in murine models [6]. Since *HOXB4* levels are known to be regulated by epigenetic mechanisms, particularly during development, and since epigenetic deregulation plays a major role in the pathogenesis of myeloid disorders, we asked whether the expression of miR-10a and *HOXB4* were also under epigenetic control. Using myeloid cell lines, we demonstrated that CpG demethylation and histone acetylation were both necessary for optimal transcription of both genes in response to retinoic acid. However, we observed differences in the sensitivity to various epigenetic modifiers in different cell lines. Mainly, OCI-AML3 was insensitive to DEOX action, possibly because of an overall decrease in DNA methylation in this *DNMT3A*-mutated cell line. The increased expressions of *HOXB4* and miR-10a were not always strictly parallel in all cell lines, suggesting that fine mechanisms may regulate differently their transcription or the stability of the transcripts. However, in all cases, chromatin opening by CpG demethylation and histone acetylation favors *HOXB4* and miR-10a overexpression, spontaneously or in response to retinoic acid.

Having demonstrated an epigenetic co-regulation of miR-10a and *HOXB4* expression in myeloid cell lines and a possible difference according to mutational status, we wondered whether the *HOXB4*/miR-10a level differences in aMPN patients could be related to mutations in epigenetic regulators genes. A sequencing panel comprising the six more frequently mutated epigenetic regulators was used to characterize a hematological malignancies cohort, showing no association between mutations in *TET2*, *ASXL1*, *IDH1/2* or *EZH2* and miR-10a/*HOXB4* levels. However, an association between *DNMT3A* mutations and miR-10a/*HOXB4* levels was found reinforcing the hypothesis of an epigenetic regulation of these genes. *DNMT3A* encodes for a methyltransferase catalyzing the addition of a methyl group to the cytosine residue of CpG dinucleotides. Ley et al sequenced *DNMT3A* in 282 AML samples and discovered mutations predominantly clustered at amino acid R882 [30]. *DNMT3A* mutants show reduced enzymatic activity resulting in decreased DNA methylation in several genomic regions including the *HOX* locus, suggesting that such a mechanism could involve the *HOXB4* and miR-10a locus [9, 30, 31]. Several groups determined the miRNA signature in normal karyotype AML patients harboring *NPM1* mutations and highlighted a strong upregulation of miR-10a and miR-10b [16, 32]. All these data were in favor of *NPM1* mutation explaining miR-10a overexpression, but it could also be hypothesized that miR-10a overexpression may be due to mutations in *DNMT3A*, strongly associated with *NPM1* mutations in AML [33]. Accordingly, OCI-AML3 cells expressing levels of miR-10a similar to *NPM1*-mutated AML primary cells also carry a *DNMT3A* R882 mutation, reinforcing this hypothesis [34]. This hypothesis was further explored in primary CD34^+^ cells. As previously shown for *HOXB4* [25], expression of the R882H mutant DNMT3A was associated with a higher expression of miR-10a as compared to WT DNMT3A expression condition. Altogether, these data support an epigenetic co-regulation of miR-10a and *HOXB4* expression in myeloid malignancies. However, the lack of strict correlation between *DNMT3A* mutation and *HOXB4*/miR-10a overexpression suggest that other regulatory mechanisms are also at play.

Having established that miR-10a and *HOXB4* were both overexpressed in aMPN patients, we investigated what the consequences of this overexpression were. The role of *HOXB4* in stem cell expansion during hematopoiesis is well documented [26–28], so we explored the role of miR-10a over-expression. Indeed, this microRNA has been described as a regulator of cell proliferation and differentiation in different models [12, 13, 29]. Moreover, Georgantas et al have predicted miR-10a as one of the 33 microRNAs involved in hematopoiesis regulation [35]. However, even though we tested different cellular models, including cell lines and primary cells, different overexpression modalities (lentiviral infections, oligonucleotide transfections) and studied different stages of hematopoiesis (from stem cells to late progenitors), we failed to demonstrate any functional effect in normal hematopoietic progenitor proliferation, differentiation or self-renewal in spite of expression levels of miR-10a similar to those observed in aMPN patients. We cannot exclude that miR-10a overexpression induces very specific modification of hematopoietic cell properties not assessed in this study, or a role restricted to malignant cells. However, our data suggest that miR-10a overexpression is a marker of the transcriptional activity at this locus without specific functional effect. Most of the abnormal aMPN phenotype could be more likely due to the overexpression of *HOXB4*, a hypothesis consistent with a recent study arguing for a bystander role of miR-10a in this context [36].

## Conclusion

In conclusion, the main dysregulated micro-RNA observed in our cohort of aMPN patients points to a “marker” micro-RNA, miR-10a, the expression of which parallels that of *HOXB4*. This dysregulation is probably linked to an epigenetic mechanism. Most of the functional consequences of this abnormality seem to be related to *HOXB4* rather than miR-10a expression.

## Additional files

Additional file 1: Material and Methods. (DOCX 19 kb)

Additional file 2: **Table S1.** Characteristics of patients with atypical myeloproliferative neoplasms (*n* = 18). **Table S2.** Characteristics of patients with hematological malignancies (*n* = 39). (DOC 136 kb)

Additional file 3: **Figure S1.** (A) Expression of HOXB4 in OCI-AML3 according to treatment by 5-aza-2’deoxycytidine (DEOX), valproic acid (VPA), and retinoic acid (RA). OCI-AML3 were treated with 2 μM DEOX and/or 1 mM VPA for 48 h and/or 2 μM RA for 4 h. qRT-PCR measured the relative expression of *HOXB4* with *TUBA1C* and *RPLP0* both used as control genes. The graphs show fold induction of gene expression by treatment over DMSO control with mean ± SEM (*n* = 3). *, *p* < 0.05; **, *p* < 0.01; ***, *p* < 0.001; NS, non-significant. (B) HOXB4 and miR-10a basal expression in myeloid cell lines. The relative expression miR-10a (right panel) and *HOXB4* (left panel) was assessed by qRT-PCR as in Fig. 2. The graphs show the mean ± SEM (*n* = 3). **Figure S2.** Impact of miR-10a overexpression on cell proliferation. K562 (top) and U937 (bottom) cells were transfected with a lentiviral vector carrying the pri-miR-10a precursor or the empty vector. Cell proliferation was evaluated by MTT assay. Results are expressed as mean +/− SEM (*n* = 4). **Figure S3.** Functional consequences of miR-10a overexpression. CD34+ cells purified from cord blood were transfected with a miR-10a or a scramble control and grown in complete medium containing SCF, TPO and Flt3-L. (A) Overexpression of miR-10a was assessed by qRT-PCR on CD34+ cells 4 days after transfection. Results are expressed as the expression of miR-10a in one representative experiment, normalized on *RNU6–1* expression and compared to K562 cell line (*n* = 2). (B) Cells were counted at different times after transfection. Results are expressed as fold increase after stimulation (*n* = 2). (C) After 7 days of culture, the expression of differentiation markers was assessed by flow cytometry. (D) After 7 days of liquid culture, cells were implanted in semi-solid medium in presence of EPO (for erythroid colonies) or G-CSF (for myeloid colonies). The graph represents the mean colony number obtained for 1000 cells (*n* = 2). **Figure S4.** Effect of miR-10a overexpression on long term stem cell self-renewal. Cells obtained from primary engraftment experiments were re-injected in mice. Differentiation markers expression on human transduced cells was assessed by flow cytometry 12 weeks after as in Fig. 5 (*n* = 3). (DOC 1017 kb)

## Abbreviations

AML: Acute myeloid leukemia
aMPN: Atypical myeloproliferative neoplasms
BMMC: Bone marrow mononuclear cells
CML: Chronic Myeloid Leukemia
CMML: Chronic MyeloMonocytic Leukemia
Cy3: Cyanine-3
DEOX: 5-aza-2’deoxycytidine
FDR: False discovery rate
HD: Healthy donor
LTC-IC: Long term culture-initiating cells
MDS: Myelodysplastic syndromes
MPN: Myeloproliferative neoplasms
NSG: NOD.Cg-Prkdscid Il2rgtm1Wjl/SzJ
OD: Optical density
RA: Retinoic acid
RHL: Reactive hyperleukocytosis
SAM: Significance analysis of microarray
uMPN/MDS: Unclassified myeloproliferative neoplasm/myelodysplastic syndrome
VPA: Valproic acid
