# Supplementary material for: MiR-10a and HOXB4 are overexpressed in atypical myeloproliferative neoplasms
Source: BMC Cancer. 2018 Nov 12;18:1098. doi: 10.1186/s12885-018-4993-2 (PMC6233495; doi:10.1186/s12885-018-4993-2)
Supplement: Supplementary file 1 — Material and Methods. (DOCX 19 kb) [file 12885_2018_4993_MOESM1_ESM.docx]

**DNA and RNA extraction**

DNA was purified with QIAamp DNA blood mini kit (Qiagen). RNA was prepared using Trizol reagent (Invitrogen). For miRNA microarray hybridization, after chloroform extraction, 1.5 volumes of 100% ethanol were added to the upper aqueous phase. The mix was deposited onto a RNeasy column (Qiagen), and was further processed according to manufacturer instructions.

**Microarray hybridization and processing**

To hybridize Cy-3 labeled RNAs onto Human miRNA Microarray V2 (Agilent Technologies), the slides were washed as recommended by the manufacturer, and scanned on an Agilent G2565CA scanner, at 5 microns resolution and using the 20-bit scan mode. Images were processed with Feature Extraction (version 10.7). Data were quantile-normalized for inter-array comparisons and analyzed using the “BRB-Array Tools” package (version 4.2.0, <http://linus.nci.nih.gov/BRB-ArrayTools.html>). Genes that were differentially expressed among the groups were identified using a F-test (Class Comparison Between groups of arrays Package, BRB-Array Tools).

**Genotyping**

The AmpliSeq panel consists of two primer pools that target the mentioned exons plus 10 to 20 bp of intronic flanking sequences. In order to amplify each library 4 μL of 5X Ion AmpliSeq™ HiFi mix, 10 μL of 2X Ion AmpliSeq™ primer pool (two of them in separate wells for each sample), 10 ng of genomic DNA per reaction (2 μL of 5 ng/μL stock), and 4 μL of nuclease free water were mixed together. The following temperature profile was applied to the final 20 μL of PCR mixture: 99°C for 2 min; 99°C for 15 sec, 60°C for 4 min (23 cycles); with a final hold at 10°C. Then primer sequences were partially digested, and adapters and barcodes ligated to the amplicons as described in Ion AmpliSeq™ library preparation manual. Each library was marked with a unique adapter provided in Ion Xpress™ barcode adapters 1–96 Kit (Life Technologies). Purified libraries were quantified with the Qubit^®^ 2.0 fluorimeter (Life Technologies) using the Qubit^®^ dsDNA HS assay kit, diluted to ~100 pmol/L and combined in equimolar proportion. Freshly prepared library stock dilutions were used on the same day for the preparation of enriched, template-positive ion sphere particles (ISPs). Automated protocols were run on the Ion OneTouch™ 2 System and the Ion OneTouch™ ES Instrument (Life Technologies) according to the version of the user guide and using the 200 bp chemistry kits. All barcoded samples were sequenced on the PGM (Life Technologies) with 314 chips taking up to 8 samples on a single chip per sequencing run.

**Cell culture and pharmacological agents**

Human leukemia cell lines, U937, KG1a OCI-AML3 and K562 (DSMZ) were cultured in RPMI 1640 medium containing 10% heat-inactivated fetal bovine serum (FBS), 1% L-glutamine, 100 U/mL penicillin, and 100 mg/mL streptomycin at 37°C in 5% CO_2_. CD34^+^ cells were purified from cord blood, obtained from normal full-term deliveries after signed informed consent, by immunomagnetic positive selection using EasySep™ (Stemcell Technologies, Grenoble, France) according to the manufacturer’s instructions. Only batches with purity > 90% as assessed by flow cytometry using CD34-PE (eBiosciences, Paris, France), were used for subsequent experiments. For liquid cultures, primary CD34^+^ cells were grown at 37°C in Stem Span SFEM (Stemcell Technologies, Grenoble, France) supplemented by SCF, FLT3-L and TPO at 50 ng/mL each. For clonogenic capacity assays, 400 to 10 000 cells were seeded in MethoCult^®^ H4534 Classic without EPO (Stemcell technologies) in presence of G-CSF (20 ng/mL) or EPO (3 U/mL). In LTC-IC assays, 25 000 cells were cultured on MS5 cells in Myelocult H5100 (Stemcell Technologies, Grenoble, France) supplemented by 10^-6^ M of hydrocortisone for 5 weeks. For all *in vitro* functional assays, transduced cells were selected by culture in presence of 10 μM puromycin.

**Lentiviral vectors and transfection**

Two infections using MOI from 20 to 45 have been performed at 12h interval. Control oligonucleotide and synthetic miR-10a were transfected into primary cells using the Amaxa human CD34^+^ cells Nucleofector kit (Lonza) according to manufacturer’s instructions.

**Real-time RT-qPCR**

For *HOXB4* mRNA quantification, qRT-PCR was performed with following primers: sense 5’-AACTCAAACTATGTCGACCCCAAG and antisense 5’-TACCCGGGCGAGTGGTC. For miR-10a-5p quantification, qRT-PCR was performed with the following primers: Universal 5’-GACGAGCTGCCTCAGTC and miR-10a 5’TACCCTGTAGATCCGAATTTG. Data obtained were analyzed by the 2^-∆∆Ct^ method. The reference genes were both *TUBA1C* and *RPLP0* for *HOXB4* and *RNU6-1* for miR10-a.

**Flow cytometry analysis**

Cellular suspensions in PEB (PBS buffer, Na2EDTA 2 mM, Bovine Serum Albumin 1%) were incubated with following fluorochrome-labelled antibodies: CD45-eFluor^®^450, CD41-PC7, CD3-PerCPCy5.5, CD235a-eFluor^®^450, CD19-PC7, CD33-PerCPCy5.5 (eBiosciences), anti-CD34-PE, CD38-APC, CD36-PE, CD14-APC (Biolegend, San Diego, CA) or isotype matched controls for 30 min at 4°C in the dark. After a PEB wash, cells were analyzed on a FACS CANTO II (Beckton Dickinson, San Jose, CA, USA). Data were analyzed with FacsDiva software.

**Proliferation assay**

Cell proliferation was assessed *in vitro* on transduced K562 and U937 cell lines using Cell Titer 96 Aqueous One Solution Cell Proliferation Assay (Promega). One hundred thousand cells were harvested in 100 μL of medium. Every day, 20 μL of reactant was added to the cells. Three hours after incubation at 37°C, optical density (OD) were measured at 490 nm. Resuls are expressed as a relative growth corresponding to the OD determined every day on OD observed at day 0.

**Mouse transplantations**

Mice were pre-conditioned 48h and 24h before engraftment with intra-peritoneal injection of 20 mg/kg of busulfan. CD34^+^ cells were thawed and transduced in StemSpan SFEM supplemented by SCF, FLT3-L, TPO (at 50 ng/mL each) and IL3 at 10 ng/mL. 10^5^ cells were then injected in the retro-orbital sinus. After 12 weeks, mice were sacrificed by cervical dislocation and the bone marrow was flushed from femurs with IMDM supplemented by FBS 1%. Red cells were lysed in an ammonium chlorhydrate buffer and then washed twice in IMDM/FBS 1% before analysis. For secondary engraftment experiments half of the cells obtained after sacrifices were re-injected into secondary NSG mouse recipients.
