# Supplementary material for: MiR-10a and HOXB4 are overexpressed in atypical myeloproliferative neoplasms
Source: BMC Cancer. 2018 Nov 12;18:1098. doi: 10.1186/s12885-018-4993-2 (PMC6233495; doi:10.1186/s12885-018-4993-2)
Supplement: Supplementary file 2 — Table S1. Characteristics of patients with atypical myeloproliferative neoplasms (n = 18). Table S2. Characteristics of patients with hematological malignancies (n = 39). (DOC 136 kb) [file 12885_2018_4993_MOESM2_ESM.doc]

**Supplementary Tables**

**Supplementary Table 1: Characteristics of patients with atypical myeloproliferative neoplasms (n=18)**

|  | **Sex** | **Age** | **Organomegaly** | **WBC**  **G/L** | **ANC**  **G/L** | **MM**  **%** | **Blast**  **%** | **Bone marrow** | **Karyotype** | **Diagnosis** | **aMPN anomalies*** | **Epigenetic modifiers anomalies**** |
| --- | --- | --- | --- | --- | --- | --- | --- | --- | --- | --- | --- | --- |
| **1** | M | 47 | none | 62.0 | 50.0 | 0 | 0 | GH, DG | normal | MPNu | NA | NA |
| **2** | M | 65 | hepatosplenomegaly | 20.0 | 19.0 | 2 | 0 | GH, DG | normal | MPNu | none | NA |
| **3** | M | 62 | splenomegaly | 142.0 | 130.0 | 8 | 0 | GH, DG | normal | aCML | SETBP1 I871I | NA |
| **4** | M | 77 | none | 41.0 | 32.4 | 10 | 1 | GH, DG | normal | aCML | none | NA |
| **5** | F | 65 | none | 24.0 | 17.0 | 18 | 2 | DG | normal | aCML | SETBP1 G870D | NA |
| **6** | M | 75 | splenomegaly | 130.0 | 105.0 | 10 | 4 | GH | normal | CNL | CSF3R T618I, SETBP1 G870S | NA |
| **7** | M | 87 | none | 43.0 | 17.0 | 7 | 2 | DG, DM | NA | aCML | SETBP1 E870K | NA |
| **8** | M | 68 | none | 48.0 | 40.0 | 0 | 0 | GH, DG | i(17q10) | CNL | CSF3R T618I | NA |
| **9** | F | 73 | hepatosplenomegaly | 58.0 | 37.0 | 6 | 2 | GH, DG | normal | aCML | none | NA |
| **10** | M | 79 | NA | 82.0 | 53.3 | 22 | 3 | GH, DG | t(1;3) | aCML | none | NA |
| **11** | M | 85 | splenomegaly | 19.3 | 11.8 | 13 | 3 | GH, DG | normal | aCML | none | NA |
| **12** | M | 72 | splenomegaly | 39.0 | 25.2 | 15 | 4 | GH, DG | normal | aCML | none | NA |
| **13** | F | 66 | none | 25.9 | 18.1 | 4 | 0 | GH | normal | MPNu | NA | ASXL1 c.1934dupG, EZH2 c.1613C>T |
| **14** | M | 78 | none | 24.0 | 18.5 | 9 | 2 | GH, DM | normal | MPNu | none | none |
| **15** | M | 69 | NA | 121.0 | 87.1 | 21 | 1 | GH, DG | normal | aCML | none | ASXL1 c.1934dupG |
| **16** | M | 82 | hepatosplenomegaly | 86.0 | 70.0 | 13 | 1 | GH | normal | CNL | none | none |
| **17** | F | 82 | splenomegaly | 62.4 | 7.5 | 13 | 0 | GH, DG | del(7q) | aCML | none | TET2 c.1872delA |
| **18** | M | 43 | hepatosplenomegaly | 80.0 | 58.0 | 7 | 1 | GH, DG | normal | aCML | none | DNMT3A c.2192T>C, IDH2 c.419G>A |

WBC: white blood cell count, ANC: absolute neutrophil count, MM: metamyelocytes and myelocytes, MPNu: myeloproliferative neoplasm unclassifiable, aCML: atypical chronic myeloid leukemia, CNL: chronic neutrophilic leukemia, DM: dysmegakaryopoiesis, DG: dysgranulopoiesis, GH: granulocytic hyperplasia, NA: not available, * among CSF3R and SETBP1, ** among ASXL1, DNMT3A, EZH2, IDH1, IDH2, TET2

**Supplementary Table 2: Characteristics of patients with hematological malignancies (n=39)**

|  | **Sex** | **Age** | **Clinical** | **WBC**  **G/L** | **ANC**  **G/L** | **MM**  **%** | **Blast**  **%** | **Bone marrow** | **Karyotype** | **Diagnosis** | **MPN anomalies*** | **Epigenetic modifiers anomalies**** |
| --- | --- | --- | --- | --- | --- | --- | --- | --- | --- | --- | --- | --- |
| **1** | F | 69 | SM | 3.3 | 1.7 | 2 | 0 | GH | NA | PMF | none | ASXL1 c.3449G>T |
| **2** | M | 55 | None | 7.2 | 4.8 | 0 | 0 | MF | NA | PMF | CALR c.1101_1161del | ASXL1 c.2957A>G |
| **3** | M | 79 | SM | 1.0 | 0.8 | 0 | 0 | MF | NA | PMF | JAK2 V617F (0.5%) | none |
| **4** | M | 86 | SM | 17.5 | 8.4 | 0 | 0 | MF | normal | PMF | none | none |
| **5** | F | 88 | None | 57.0 | 48.0 | 8 | 2 | MF | normal | PMF | JAK2 V617F (0.2%) | ASXL1 c.1934dupG, TET2 c.5636A>T |
| **6** | M | 50 | SM | 7.6 | 5.3 | 14 | 1 | Hyperplasia | normal | PMF | none | none |
| **7** | M | 57 | SM | 55.6 | 33.6 | 0 | 0 | NA | normal | PMF | JAK2 V617F (63%) | ASXL1 c.1934dupG, TET2 c.3922A>T |
| **8** | M | 71 | HSM | 10.7 | 8.5 | 3 | 0 | MF | NA | PMF | JAK2 V617F (44%) | TET2 c.4011dupT |
| **9** | M | 10 | SM | 6.2 | 4.5 | 4 | 4 | MF | del(20q) | PMF | JAK2 V617F (42%) | none |
| **10** | F | 19 | SM | 4.5 | 2.0 | 0 | 0 | MF | NA | PMF | JAK2 V617F (39%) | none |
| **11** | M | 59 | None | 8.3 | 3.8 | 11 | 3 | MF | normal | PMF | none | EZH2 c.553G>C |
| **12** | M | 50 | SM | 5.9 | 4.7 | 0 | 0 | MF | NA | PMF | none | none |
| **13** | M | 79 | SM | 15.1 | 11.3 | 0 | 0 | NA | NA | PMF | none | none |
| **14** | F | 58 | None | 14.0 | 9.9 | 4 | 0 | MF | del(13q) | PMF | JAK2 V617F (37%) | none |
| **15** | F | 21 | HSM | 11.0 | 7.0 | 9 | 0 | MF | normal | PMF | JAK2 V617F (NA) | none |
| **16** | M | 76 | NA | 10.0 | 6.4 | 11 | 0 | NA | NA | PMF | CALR c.1092_1143del | ASXL1 c.2633_2634insC |
| **17** | F | 89 | None | 13.0 | 10.5 | 0 | 0 | MF | NA | PMF | MPL W515K | none |
| **18** | M | 54 | SM | 52.2 | 24.6 | 33 | 1 | MF | normal | PMF | JAK2 V617F (77%) | ASXL1 c.2769_2776del |
| **19** | M | 50 | SM | 51.0 | 19.0 | 7 | 0 | GH | normal | PMF | JAK2 V617F (55%) | ASXL1 c1934dupG, TET2 c.4260A>T |
| **20** | M | 50 | HSM | 25.7 | 20.0 | 14 | 3 | GH | normal | CMML | none | ASXL1 c1934dupG |
| **21** | M | 62 | SM | 19.8 | 8.5 | 28 | 1 | MF | normal | CMML | JAK2 V617F (50%) | DNMT3A c.2645G>A |
| **22** | M | 81 | SM | 40.0 | 31.4 | 0 | 0 | MF | NA | PMF | JAK2 V617F (71%) | TET2 c.2081delT |
| **23** | M | 69 | HSM | 9.5 | 8.3 | 0 | 0 | MF | NA | PMF | JAK2 V617F (84%) | none |
| **24** | M | 78 | SM | 8.8 | 6.6 | 0 | 0 | MF | NA | PMF | CALR c.1092_1143del | TET2 c.2145_2146del |
| **25** | F | 60 | SM | 8.0 | 4.0 | 0 | 0 | MF | normal | PMF | CALR c.1092_1143del | DNMT3A c.2645G>A |
| **26** | M | 75 | SM | 5.0 | 0.0 | 6 | 0 | MF | del(13q) | PMF | JAK2 V617F (50%) | IDH1 c.395G>A |
| **27** | F | 79 | None | 1.3 | 0.6 | 0 | 0 | 25% blasts | normal | AML | NA | NPM1 wild type, DNMT3A c.2645G>A |
| **28** | M | 77 | None | 1.4 | 0.1 | 0 | 6 | 95% blasts | normal | AML | NA | NPM1 wild type, DNMT3A c.2645G>A |
| **29** | M | 65 | None | 40 | 8.2 | 0 | 32 | 42% blasts | normal | AML | NA | NPM1 wild type, DNMT3A c.2645G>A |
| **30** | M | 61 | None | 4.8 | 1.2 | 0 | 0 | 46% blasts | +11 | AML | NA | NPM1 wild type, DNMT3A c.2645G>A |
| **31** | F | 65 | SM | 9.3 | 4.9 | 12 | 6 | MF | NA | PMF | CALR c.1092_1143del | DNMT3A c.2645G>A, TET2 c.5705A>T, ASXL1 c.2254dupG |
| **32** | M | 42 | SM | 8.7 | 2.4 | 0 | 0 | EH | NA | MPNEo | FIP1L1-PDGFRA | none |
| **33** | H | 82 | NA | 11.5 | 6.0 | 0 | 0 | NA | NA | ET | JAK2 V617F (43%) | TET2 c.3058C>T |
| **34** | H | 57 | None | 23.8 | 15.5 | 18 | 0 | GH | t(5;10) | MPNEo | PDGFRB | none |
| **35** | F | 90 | None | 31.0 | 25.8 | 1 | 0 | NA | NA | uMPN | JAK2 V617F (52%) | SRSF2 c.284C>A, TET2 c.3860T>C |
| **36** | H | 72 | SM | 25.0 | 16.0 | 5 | 1 | DM | NA | PMF | JAK2 V617F (93%) | RUNX1 c.587C>T, SRSF2 c.284C>G, TET2 c.3929A>T |
| **37** | F | 75 | None | 33.8 | 29 | 4 | 0 | MF | NA | PPV-PMF | JAK2 V617F (95%) | DNMT3A c.976C>T |
| **38** | H | 75 | None | 9.01 | 6.62 | 0 | 0 | NA | NA | PV | JAK2 V617F | DNMT3A c.2299delA |
| **39** | H | 66 | NA | 15.5 | 10.9 | 0 | 0 | NA | NA | ET | JAK2 V617F (37%) | DNMT3A c.2195T>C |

WBC: white blood cell count, ANC: absolute neutrophil count, MM: metamyelocytes and myelocytes, PMF: primary myelofibrosis, AML: acute myeloid leukemia, MPNEo: Myeloid/lymphoid neoplasms with eosinophilia, CMML: chronic myelomonocytic leukemia, SM: Splenomegaly, HSM: Hepatosplenomegaly, GH: granulocytic hyperplasia, EH: eosinophilic hyperplasia, DM: dysmegakaryopoiesis, MF: myelofibrosis, NA: not available, * among JAK2, CALR, MPL and BCR-ABL1, ** among ASXL1, DNMT3A, EZH2, IDH1, IDH2, TET2
