# Supplementary figures and images for: MiR-10a and HOXB4 are overexpressed in atypical myeloproliferative neoplasms
Source: BMC Cancer. 2018 Nov 12;18:1098. doi: 10.1186/s12885-018-4993-2 (PMC6233495; doi:10.1186/s12885-018-4993-2)

**A**

**B**

Supplementary Figure 1


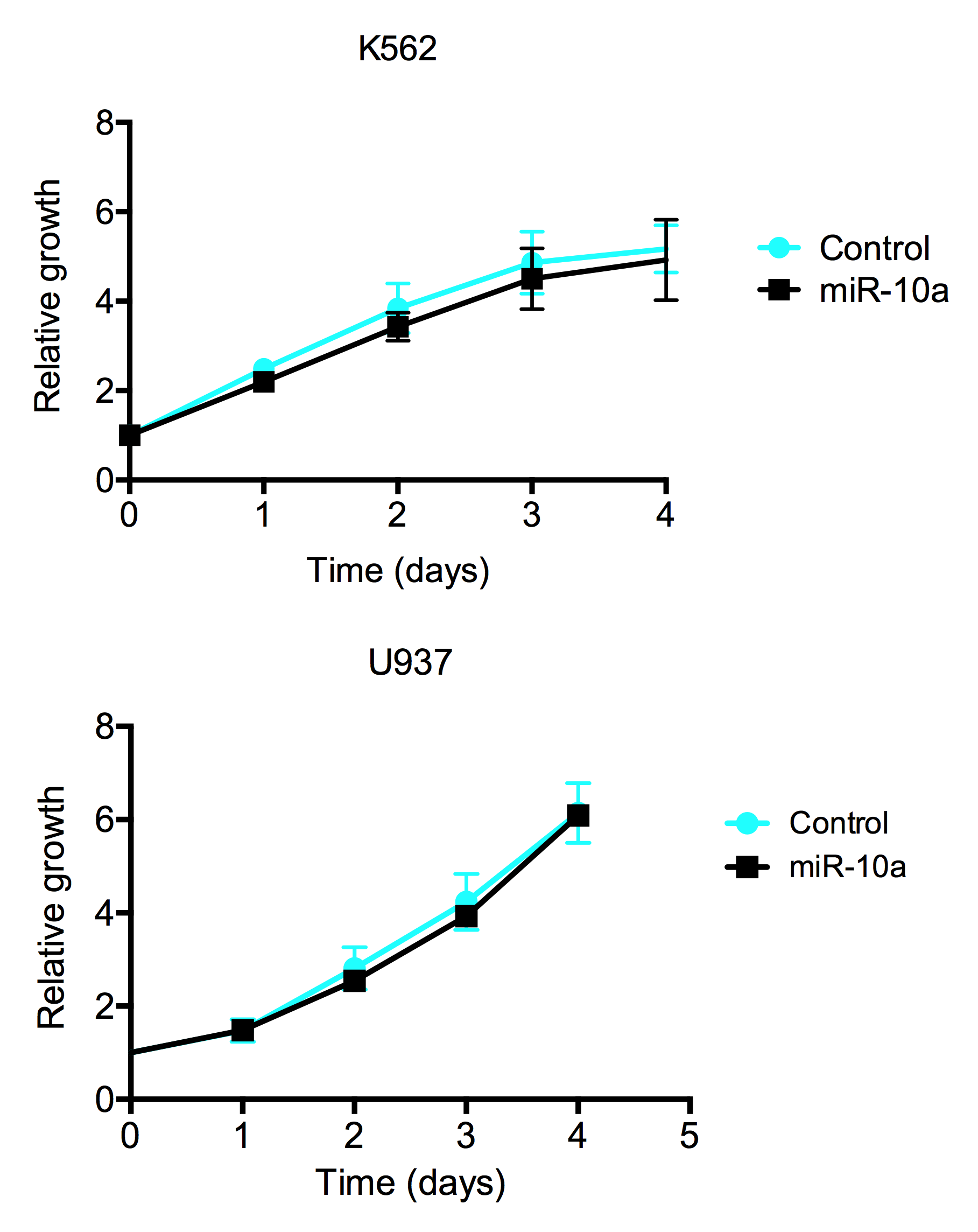


Supplementary Figure 2


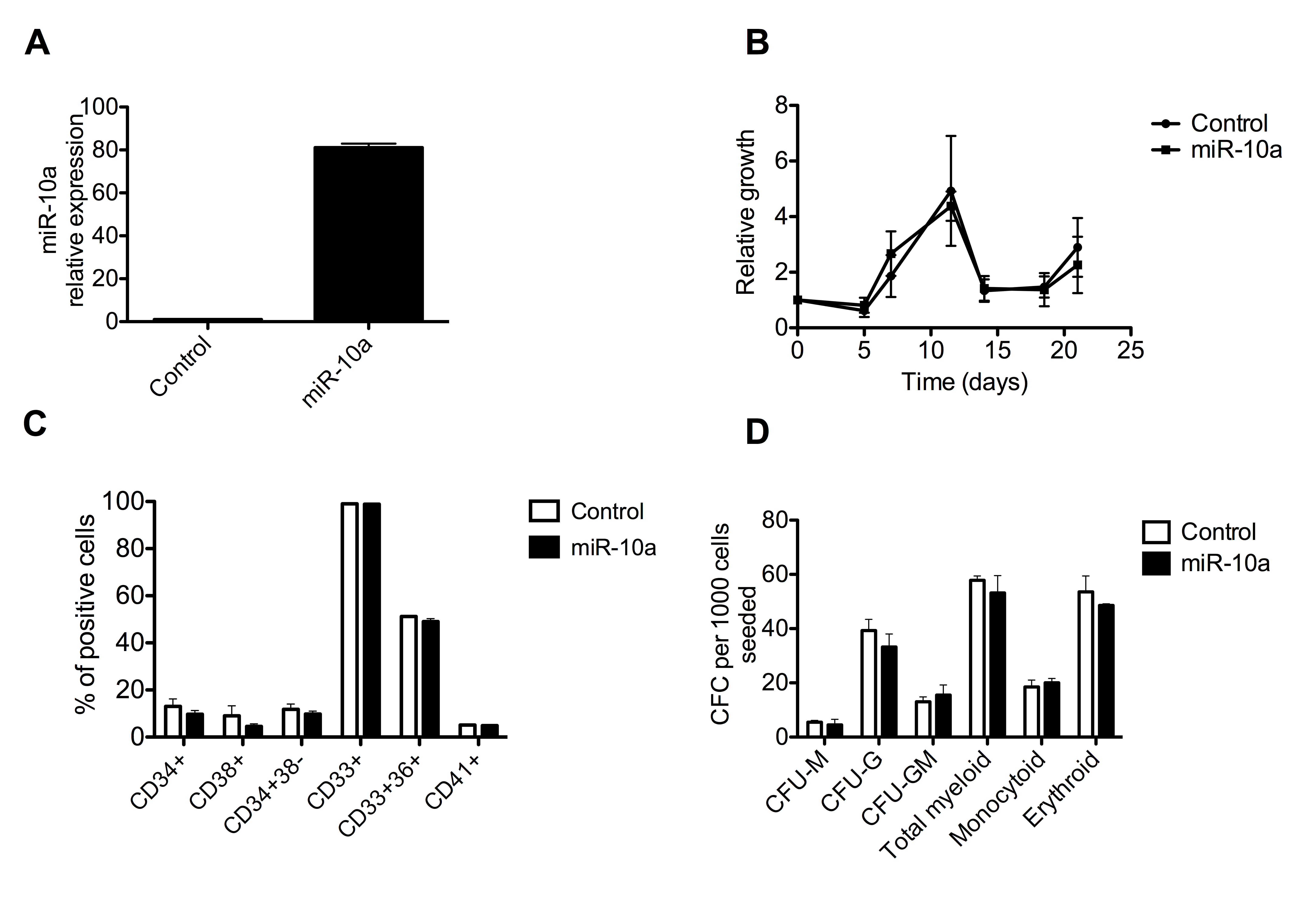


Supplementary Figure 3

Supplementary Figure 4

Supplement: Supplementary file 3 — Figure S1. (A) Expression of HOXB4 in OCI-AML3 according to treatment by 5-aza-2’deoxycytidine (DEOX), valproic acid (VPA), and retinoic acid (RA). OCI-AML3 were treated with 2 μM DEOX and/or 1 mM VPA for 48 h and/or 2 μM RA for 4 h. qRT-PCR measured the relative expression of HOXB4 with TUBA1C and RPLP0 both used as control genes. The graphs show fold induction of gene expression by treatment over DMSO control with mean ± SEM (n = 3). *, p < 0.05; **, p < 0.01; ***, p < 0.001; NS, non-significant. (B) HOXB4 and miR-10a basal expression in myeloid cell lines. The relative expression miR-10a (right panel) and HOXB4 (left panel) was assessed by qRT-PCR as in Fig. 2. The graphs show the mean ± SEM (n = 3). Figure S2. Impact of miR-10a overexpression on cell proliferation. K562 (top) and U937 (bottom) cells were transfected with a lentiviral vector carrying the pri-miR-10a precursor or the empty vector. Cell proliferation was evaluated by MTT assay. Results are expressed as mean +/− SEM (n = 4). Figure S3. Functional consequences of miR-10a overexpression. CD34+ cells purified from cord blood were transfected with a miR-10a or a scramble control and grown in complete medium containing SCF, TPO and Flt3-L. (A) Overexpression of miR-10a was assessed by qRT-PCR on CD34+ cells 4 days after transfection. Results are expressed as the expression of miR-10a in one representative experiment, normalized on RNU6–1 expression and compared to K562 cell line (n = 2). (B) Cells were counted at different times after transfection. Results are expressed as fold increase after stimulation (n = 2). (C) After 7 days of culture, the expression of differentiation markers was assessed by flow cytometry. (D) After 7 days of liquid culture, cells were implanted in semi-solid medium in presence of EPO (for erythroid colonies) or G-CSF (for myeloid colonies). The graph represents the mean colony number obtained for 1000 cells (n = 2). Figure S4. Effect of miR-10a overexpression on long term stem c [file 12885_2018_4993_MOESM3_ESM.doc]
